# Chemically-Defined, Xeno-Free, Scalable Production of hPSC-Derived Definitive Endoderm Aggregates with Multi-Lineage Differentiation Potential

**DOI:** 10.3390/cells8121571

**Published:** 2019-12-04

**Authors:** Anais Sahabian, Malte Sgodda, Ortwin Naujok, Rabea Dettmer, Julia Dahlmann, Felix Manstein, Tobias Cantz, Robert Zweigerdt, Ulrich Martin, Ruth Olmer

**Affiliations:** 1 Leibniz Research Laboratories for Biotechnology and Artificial Organs (LEBAO), REBIRTH - Center for Regenerative Medicine, Hannover Medical School, 30625 Hannover, Germany; dahlmann.julia@mh-hannover.de (J.D.); manstein.felix@mh-hannover.de (F.M.); Zweigerdt.Robert@mh-hannover.de (R.Z.); martin.ulrich@mh-hannover.de (U.M.); 2 Biomedical Research in Endstage and Obstructive Lung Disease Hannover (BREATH), German Center for Lung Research (DZL), Hannover Medical School, 30625 Hannover, Germany; 3 Department of Gastroenterology, Hepatology and Endocrinology and MPI-Cell and Developmental Biology, REBIRTH - Center for Regenerative Medicine, Hannover Medical School, 30625 Hannover, Germany; sgodda.malte@mh-hannover.de (M.S.); cantz.tobias@mh-hannover.de (T.C.); 4 Institute of Clinical Biochemistry, REBIRTH - Center for Regenerative Medicine, Hannover Medical School, 30625 Hannover, Germany; naujok.ortwin@mh-hanover.de (O.N.); dettmer.rabea@mh-hannover.de (R.D.)

**Keywords:** pluripotent stem cells, definitive endoderm differentiation, suspension culture, multi-lineage potential, cryopreservation

## Abstract

For the production and bio-banking of differentiated derivatives from human pluripotent stem cells (hPSCs) in large quantities for drug screening and cellular therapies, well-defined and robust procedures for differentiation and cryopreservation are required. Definitive endoderm (DE) gives rise to respiratory and digestive epithelium, as well as thyroid, thymus, liver, and pancreas. Here, we present a scalable, universal process for the generation of DE from human-induced pluripotent stem cells (hiPSCs) and embryonic stem cells (hESCs). Optimal control during the differentiation process was attained in chemically-defined and xeno-free suspension culture, and high flexibility of the workflow was achieved by the introduction of an efficient cryopreservation step at the end of DE differentiation. DE aggregates were capable of differentiating into hepatic-like, pancreatic, intestinal, and lung progenitor cells. Scale-up of the differentiation process using stirred-tank bioreactors enabled production of large quantities of DE aggregates. This process provides a useful advance for versatile applications of DE lineages, in particular for cell therapies and drug screening.

## 1. Introduction

Various acquired and inherited diseases of the lung, liver, intestine, and pancreas are heavy burdens for the affected patients. A better understanding of normal and disease processes and cellular pathomechanisms of functional cells might provide possible new targets for medical interventions. In addition, patients could benefit from cell replacement therapies. It has been estimated that more than 10^9^ pancreatic islet cells are needed for effective treatment of type I diabetes mellitus [1]. For liver-based metabolic diseases and acute liver failure, most clinics use up to 2 × 10^8^ hepatocyte cells/kg to treat patients [2]. As these required cell numbers can scarcely be generated from autologous, primary cells, alternative cell sources for clinical applications are of great interest. Human pluripotent stem cells (hPSCs) have great potential as a universal therapeutic cell source for different diseases because they can be grown indefinitely while maintaining their potential to differentiate into the three germ layers [3]. In addition, they have been shown to be useful tools for basic research, in vitro disease modelling and high throughput drug screening assays [4,5]. In one example, 1.3 × 10^9^ differentiated cystic fibrosis patient-specific iPSCs were used to screen 42,500 compounds for modulators of CFTR activity [5].

As both the routine use of hPSC-based cell therapies and organotypic in vitro cell applications become more important [6], new systems are needed to reproducibly deliver large quantities of cells that meet the required standards of “good manufacturing practice” (GMP) [7,8]. Currently, many laboratories are still performing adherent 2D cell culture for expansion and differentiation of hPSCs. However, when it comes to large scale cell production in 2D, there are two major limitations: these systems are restricted to culture surface area and require a lot of manual work. Culturing cells in dynamic 3D suspension using platforms such as a bioreactor can overcome these restraints, as such systems have a high surface area-to-volume ratio. We and others have already shown that undifferentiated hPSCs can be maintained and expanded in suspension cultures up to clinically relevant cell numbers [1,7,9] and that further differentiation to cardiomyocytes [10] and endothelial cells [11] is applicable in this format. From a technical point of view, bioreactors allow linear upscaling processes with multi-parametric monitoring possibilities (pH, metabolics, feeding, etc.) combined with versatile options for process intervention and optimization, and for the generation of homogeneous production batches. The combination with an efficient cryopreservation protocol would further advance the process for off-the-shelf allocation of definitive endoderm (DE) as starting material for production of different DE lineages.

It has been previously shown that DE differentiation in suspension is feasible [12,13,14,15,16,17,18]. According to Yabe et al., differentiation into DE in suspension may be superior to adherent culture differentiation, since DE markers appear faster and are more strongly expressed [12]. Despite promising prospects to use suspension cultures for DE differentiation, most of these protocols do not use chemically-defined, xeno-free media. In addition, the capability of these aggregates to differentiate into most lineages of the DE has not yet been demonstrated. Considering the technical and biological requirements of industrial scale cell production, we established a straightforward protocol for chemically-defined, xeno-free generation of DE from hiPSCs and hESCs. We tested two different methods for DE generation to see if both conditions could give rise to viable DE. We first used Erlenmeyer flasks for proof-of principle purposes, and then successfully scaled up to a controlled, stirred bioreactor system. Finally, we showed that hPSC-derived DE cells maintain their multi-lineage differentiation potential after cryopreservation and thawing. This work is a valuable step towards efficient disease modeling, drug discovery, and clinical scale up of therapeutic DE cell products.

## 2. Materials and Methods

### 2.1. Stem Cell Culture 

The hESC line, HES3, and the hiPSC cell line, MHHi001-A [19], generated from CD34+ cells of a healthy patient using Sendai virus, were used for all experiments. The third cell line (MHHi006-A) was generated from a healthy patient using lenti-viral based reprogramming. Undifferentiated hPSCs were cultivated on Geltrex™ (Gibco #A1413202) using E8 medium (in house) for up to 12 passages. The cells were passaged with StemPro^®^ Accutase^®^ (Gibco #A1110501) twice a week. 

### 2.2. DE Differentiation Using CHIR99021 and Activin A (CA)

At day 1, a 90% confluent flask of hPSCs was dissociated with StemPro^®^ Accutase^®^ (Gibco #A1110501), and inoculated in 125 mL Erlenmeyer flasks (Corning #431143) containing 20 mL of E8 medium (in house) with 10 µM Y-27632 (Tocris #1254) at a cell density of 3.33 × 10^5^ cells/mL for hiPSCs and 6.67 × 10^5^ cells/mL for hESCs. Flasks were placed in an incubator on an orbital shaker (Celltron, Infors HT) rotating at 70 rotations per minute (rpm). Medium was changed every 24 h (h). For each medium change, the aggregates were collected and centrifuged (Multifuge 3SR+, Thermo) at 300 g for 2 min before placing them in fresh medium. The following day (day 0), the media was changed to RPMI 1640 (Gibco #21875034), 100 ng/mL activin A (PeproTech #120-14E), and 3 µM CHIR99021 (provided by the Institute of Organic Chemistry, Leibniz University, Hannover, Germany). On day 1, the medium was changed to 100 ng/mL activin A, and 0.8% Knockout™ Serum Replacement (KSR; Gibco #10828028) in RPMI 1640. On day 2, the medium was changed to 100 ng/mL activin A, 8% KSR in RPMI 1640. On day 3 of differentiation, aggregates were dissociated with Accutase for 3 min in a water bath at 37 °C, analyzed for DE markers, and further differentiated into liver, pancreatic, intestinal, and lung progenitor cells. Dissociated day 3 cells were also frozen in CryoStor^®^ CS10 Freezing Media (BioLife Solutions #210102).

### 2.3. DE Differentiation Using STEMDiff™ Definitive Endoderm Kit (SD)

Six days before the start of differentiation, pluripotent stem cells were passaged in flasks at a low density, 1.2 × 10^4^ cells/cm^2^, using E8 medium and 10 µM Y-27632 (Tocris #1254). Medium was changed every 24 h. At day 3, medium was changed to E8 medium and Supplement 1, supplied in the SD kit (STEMCELL Tech. #05115). On day 1 of differentiation, cells were dissociated with StemPro^®^ Accutase^®^ (Gibco #A1110501) and inoculated in shaker flasks, at a cell density of 3.33 × 10^5^ cells/mL for hiPSCs and 6.67 × 10^5^ cells/mL for hESCs, in E8 medium, E8 supplement (STEMCELL Tech. #05116), and 10 µM Y-27632. For each medium change, the aggregates were collected and centrifuged (Multifuge 3SR+, Thermo) at 300× *g* for 2 min before placing them in fresh medium. On day 0, medium was changed to STEMDiff™ Endoderm Basal Media containing Supplement MR and CJ. On day 1 and day 2, aggregates were fed with STEMDiff™ Endoderm Basal Media containing Supplement CJ only. On day 3, aggregates were dissociated and analyzed for DE markers, and also further differentiated into liver, pancreatic, intestinal, and lung progenitor cells. Dissociated cells were also frozen in CryoStor^®^ CS10 Freezing Media (BioLife Solutions #210102) at 6 × 10^6^ cells/vial.

### 2.4. Differentiation into the Hepatic Lineage

For hepatic differentiation, aggregates on day 3 of DE differentiation were adapted to hepatic differentiation media [20]. In short, the medium was changed to hepatocyte culture medium (Lonza #CC-3198) with 30 ng/mL of fibroblast growth factor 4 (FGF-4, Peprotech #100-31), 20 ng/mL of bone morphogenetic protein 2 (BMP-2, Peprotech #120-02), and 10 µM SB431542 (Sigma Aldrich #S4317), 0.5 µg/mL of secreted frizzled-related protein 5 (sFRP-5, R&D Systems #6266-SF) for 24 h in Erlenmeyer flasks rotating at 70 rpm. Aggregates were then dissociated into single cells using TrypLE (Thermo Fisher #12604013) and plated on Matrigel^®^ (Corning #356231) coated plates with a density of 45,000 cells/cm^2^ in hepatic differentiation media containing 10 µM Y-27632 (Tocris #1254). The cells were cultured for three more days with daily medium changes. On day 5 of differentiation, the medium was changed to hepatocyte culture medium supplemented with 20 ng/mL hepatocyte growth factor (HGF, Peprotech #100-39) for a further four days with daily medium change. On day 9 of differentiation, the medium was changed to hepatocyte culture medium (Lonza) with 20 ng/mL HGF (Peprotech #100-39), 10 ng/mL Oncostatin M (OSM; Peprotech #300-10) and 10 ng/mL dexamethasone (Sigma Aldrich #D4902) for an additional four days. Cells were analyzed on day 14 of differentiation.

### 2.5. Differentiation into the Pancreatic Lineage

For differentiation into pancreatic PDX1+ cells [21], aggregates were dissociated using Accutase (Capricorn #ACC-1B), counted, and seeded on plates coated with Matrigel^®^ (Corning #354277)at a density of 2.6 × 10^5^ cells/cm^2^ in Advanced RPMI 1640 medium (Gibco #12-633-012) supplemented with 1 µM all-trans retinoic acid (Sigma Aldrich #302-79-4), 0.5 µM LDN 193,189 (Selleckchem #DM-3189), 2 µM IWR-1 (Selleckchem #S7086), 5 ng/mL FGF7 (Reliatech #100-163-L), 0.5× B27 (Gibco #17-504-044), 1% L-glutamine (Sigma Aldrich #G7513), and 1% penicillin/streptomycin (Santa Cruz #sc-391048, Sigma Aldrich # S9137). 10 µM Y-27632 (Selleckchem #S1049) was added for the first 24 h. Differentiation was performed in 12-well plates and 4-well slides (SPL Life Sciences) for immunofluorescent (IF) staining. The medium was changed daily for an additional 7 days (day 10), and then harvested for qRT-PCR analysis or fixed for IF staining.

### 2.6. Differentiation into the Intestinal Lineage

A previously established protocol [22] was adapted where WNT3A was substituted with CHIR99021. On day 3 of DE differentiation, aggregates were dissociated with Accutase (Gibco #A1110501) and plated down at 2 × 10^5^ cells/cm^2^ in intestinal medium: DMEM/F12 (Gibco #11330032), 2% fetal bovine serum (PAA #A11-101), 500 ng/mL FGF4 (PeproTech #100-31), 3 µM CHIR99021 (provided by the Institute of Organic Chemistry, Leibniz University, Hannover, Germany), and 1% penicillin/streptomycin (Thermo Fisher #15140122). Medium was changed every other day until day 7, when cells were analyzed.

### 2.7. Differentiation into Lung Progenitor Cells

On day 3 of DE differentiation, aggregates were dissociated with Accutase (Gibco #A1110501) and plated at 1 × 10^5^ cells/cm^2^ for hiPSC-derived SD condition, 2.0 × 10^5^ cells/cm^2^ for hESC-derived SD condition, 2.0 × 10^5^ cells/cm^2^ for hiPSC-derived CA, and 2.57 × 10^5^ cells/cm^2^ for hESC-derived CA condition, in anterior foregut medium 1 (AFE1): lung basal medium (LBM; Knockout™ DMEM (Gibco #12660012), 5% KSR (Gibco #10828028), 1% l-glutamine (Gibco #25030081), 1% non-essential amino acids (Gibco #11140035), 1% penicillin/streptomycin (Gibco #25030081), 0.46 mM 1-thioglycerol (Sigma-Aldrich #M6145)), 3 µM dorsomorphin (Sigma-Aldrich #P5499), and 10 µM SB435142 (provided by the Institute of Organic Chemistry, Leibniz University, Hannover, Germany) with 10 µM Y-27632 (Tocris #1254). On day 5, medium was changed to anterior foregut medium 2 (AFE2): LBM containing 2 µM IWP-2 (Tocris #3533) and 10 µM SB435142. On day 7, cells in the SD condition were fed with B4CF10 medium: LBM containing 10 ng/mL BMP-4 (R&D Systems #314-BP), 3 µM CHIR99021 (provided by the Institute of Organic Chemistry, Leibniz University, Hannover, Germany), and 10 ng/mL FGF10 (R&D Systems #345-FG), while cells in the CA condition were passed with TrypLE (Thermo Fisher #12604013) at a ratio of 1:2 in B4CF10 with 10 µM Y-27632. B4CF10 medium was changed every 2–3 days and cells were collected between days 10–14 for analysis.

### 2.8. Thawing of Dissociated DE Aggregates for Further Differentiation

When differentiating cryopreserved DE cells, the same protocols as described above were used to differentiate into each lineage, with some changes to cell seeding density. For hepatic differentiation, cells were seeded at 4 × 10^4^ cells/cm^2^. For pancreatic differentiation, cells were seeded at 5.26 × 10^5^ cells/cm^2^. For intestinal differentiation, cells were seeded at 2 × 10^5^ cells/cm^2^. Media was changed the following day to remove cell debris, and then fed every other day. For lung differentiation, cells were seeded at a density of 1.8 × 10^5^ cells/cm^2^ for the SD condition and 2.85 × 10^5^ cells/cm^2^ for the CA condition. After 24 h, the cells were fed with fresh AFE1 and the same protocol was used as described in the lung progenitor differentiation section above.

### 2.9. DE Differentiation in Controlled, Stirred Bioreactor

DE differentiation in the bioreactor was performed using a DASbox Mini Bioreactor System (Eppendorf). Parallel operation comprised of two independently controlled DASbox Mini bioreactor vessels for cell culture applications equipped with an eight-blade impeller (60° pitch) optimized for hPSC expansion [9]. An overhead drive allowed for smooth agitation. Sensors for pH and DO monitoring as well as temperature control ensured a tight monitoring and control of critical process parameters. Sensors were calibrated as previously described [10]. The bioreactors were inoculated with hiPSCs at a density of 5 × 10^5^ cells/mL in E8 (in house) and 10 µM Y-27632 (Tocirs #1254) in a final culture volume of 150 mL (75 × 10^6^ cells per vessel). Cells were cultivated at 37 °C, stirred at 60 rpm, headspace aerated with 3 sl/hour with 21% O_2_ and 5% CO_2_. The same feeding protocol was applied as described in the DE differentiation section above. Differentiation into all the lineages was performed using the same protocol used for differentiation in the Erlenmeyer flask.

### 2.10. Cryosectioning and IF Staining of Day 3 Aggregates

On day 3, aggregates were placed in Tissue Tek^®^ (Sakura #SA62550-01), frozen on dry ice and stored at −80 °C. Samples were sectioned (10 µm slices) using a microtom, mounted on microscope slides, air-dried for 1 h, and stored at −80 °C. IF staining was performed as described in Supplemental Experimental Procedures. Antibodies used can be found in Supplementary Materials (Table S1). After staining procedures were performed, slides were covered with cover slips using fluorescent mounting medium (Dako #S3023).

### 2.11. Immunofluorescent Staining and Quantification

Detailed immunostaining procedures are described in the Supplemental Experimental Procedures. For quantification of hepatic differentiation, 10 grey-scale images were taken in three independent experiments. The DAPI/HNF4α positive area was evaluated using ImageJ with the threshold set between 9–12 for the left limit and 50 for the right for all images within one experiment. For intestinal, lung, and pancreatic progenitor cell quantification, 4–6 images from three independent experiments were quantified using the plugin image-based tool for counting nuclei (ITCN) on ImageJ.

### 2.12. Flow Cytometry

Flow cytometry was performed on aggregates at day 3 of DE differentiation. Aggregates were dissociated with Accutase (Gibco #A1110501), washed with FACS buffer (0.5% BSA, 3 mM EDTA, PBS), and stained with CXCR4-APC (eBioscience #17-999-42), c-Kit-PE (eBioscience #12-1178-42), and EpCam-PE (BD #347198) at the company-recommended dilution on ice for 30 min. The cells were then washed with FACS buffer and analyzed using the MACSQuant^®^ Analyzer 10. FlowJo V10 software was used to analyze the data.

### 2.13. Quantitative Real-Time PCR

Total-RNA isolation, reverse transcription, and qRT-PCR procedures are described in detail in the Supplemental Experimental Procedures.

### 2.14. Aggregate Size and Cell Density Measurement

To monitor aggregate formation and diameters, four independent light microscopic images were captured for each sample (Axiovert A1; Zeiss) followed by diameter analysis via ImageJ. Mean diameters represent arithmetic averages from 76–470 single aggregates. Cell density measurements were taken with Vi-Cell™ XR Cell Viability Analyzer (Beckman Coulter).

### 2.15. Statistics

Unpaired, parametric, two-tailed *t*-test was performed on all data sets via GraphPad Prism.

## 3. Results

### 3.1. Definitive Endoderm Differentiation of Pluripotent Stem Cells in Scalable Suspension Culture

**Figure 1:**
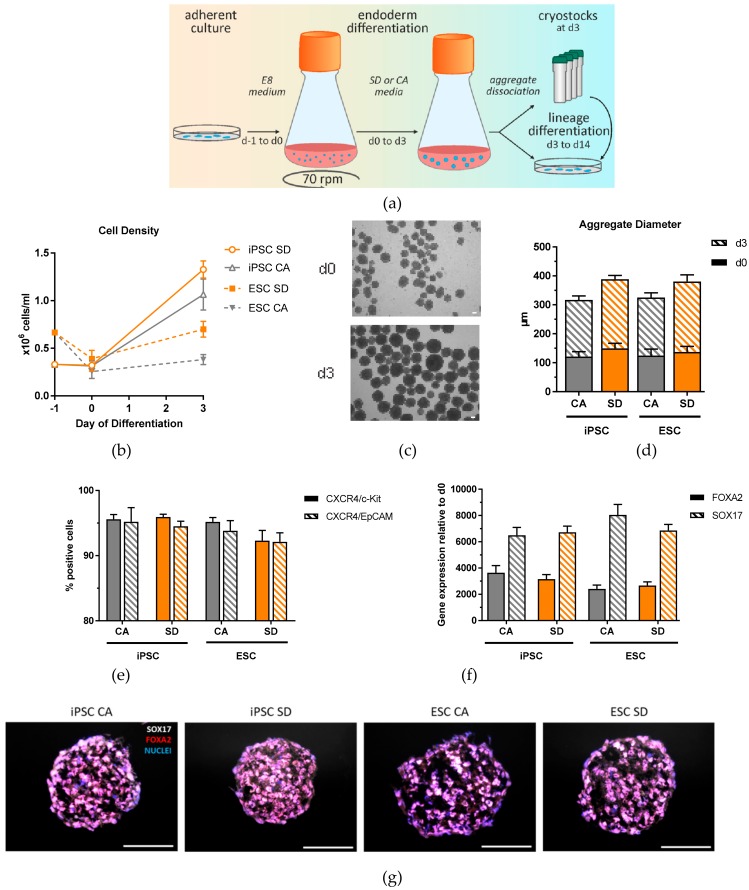
Generation and characterization of DE aggregates in Erlenmeyer flasks. (**a**) Schematic overview of definitive endoderm differentiation in suspension. (**b**) Cell density analyzed at day 0 and day 3 of differentiation (n = 4–6). (**c**) Representative brightfield image of iPSC aggregates at day 0 and day 3 of differentiation (n = 4–6). (**d**) Aggregate diameters measured at day 0 and day 3 of differentiation. (**e**) Definitive endoderm efficiency quantification based on flow cytometry analysis of CXCR4/c-Kit and CXCR4/EpCAM double positive cells at day 3 of differentiation (n = 12–15). (**f**) qRT-PCR analysis of FOXA2 and SOX17 expression at day 3 of differentiation (n = 6–9). (**g**) Immunostaining of SOX17 (white) and FOXA2 (red), and nuclear stain DAPI (blue) of DE aggregates at day 3 of differentiation (n = 3). Scale bar, 100 μm. Each value of gene expression was first normalized to the reference genes and then to day 0 undifferentiated cells. Values are represented as the mean ± SEM. All n values correspond to independent experiments.

To establish DE differentiation in dynamic suspension culture, we inoculated single hiPSCs and hESCs from adherent expansion cultures in Erlenmeyer flasks in E8 medium containing 10 µM Y-27632 (Figure 1a).

Initial experiments with the hESC line, using the same starting density as that used for the hiPSCs (3.33 × 10^5^ cells/mL), resulted in the formation of very few aggregates. Therefore, the inoculation cell density was increased to 6.66 × 10^5^ cells/mL, resulting in aggregates at a similar cell density as for hiPSCs 24 h after inoculation (day 0) (Figure 1b). On day 0, round, uniform aggregates were observed (Figure 1c) ranging from 120–150 μm in diameter (Figure 1d). These aggregates were differentiated towards DE for three days using two different culture conditions. The first condition was adapted from a protocol established by Miller et al. [23]. The medium for this condition included a low concentration of CHIR99021, a GSK3beta inhibitor which induces DE via activation of the canonical Wnt pathway, and activin A, which mimics Nodal signaling to specify DE [24]. The second condition used a commercially available kit, which has been used in many published protocols to generate DE [5,25,26,27,28,29,30]. Both conditions are chemically-defined and xeno-free, making it more easily adjustable to GMP requirements needed for future clinical cell therapy applications. By day 3 of differentiation, a moderate increase in aggregate size was observed in both cell lines and conditions (Figure 1c), with mean aggregate diameters ranging from 195–244 μm (Figure 1d), reflecting the increase in cell number to an average of 8.7 × 10^5^ cells/mL. It was observed that the aggregates from the SD condition had larger diameters than the CA condition. In addition, the cell densities from the hiPSC line were higher than those from hESCs (Figure 1b). On day 3, efficient DE generation was confirmed by expression analysis of surface antigens CXCR4, c-Kit, and EpCAM. More than 92% of cells were positive for all markers in all conditions (Figure 1e). Quantitative real time PCR (qRT-PCR) showed strong induction of DE markers *FOXA2* and *SOX17* compared to day 0 cells (Figure 1f), and homogenous expression of these markers throughout the aggregates was shown by immunofluorescence (IF) staining of aggregate cross-sections on day 3 of differentiation (Figure 1g). Another iPSC line was differentiated towards DE using this system and characterized demonstrating the robustness of this protocol and exemplifying its potential use with any hPSC line (Supplementary Materials Figure S1). Growth rates of the CA and SD conditions are significantly higher compared to adherent culture growth rate during DE differentiation (Supplementary Materials Figure S1f). Taken together, our results indicate that three hPSC lines can differentiate towards DE in Erlenmeyer flasks with high efficiency using both the CA and SD conditions.

### 3.2. Multi-Lineage Differentiation Potential of DE Aggregates

**Figure 2:**
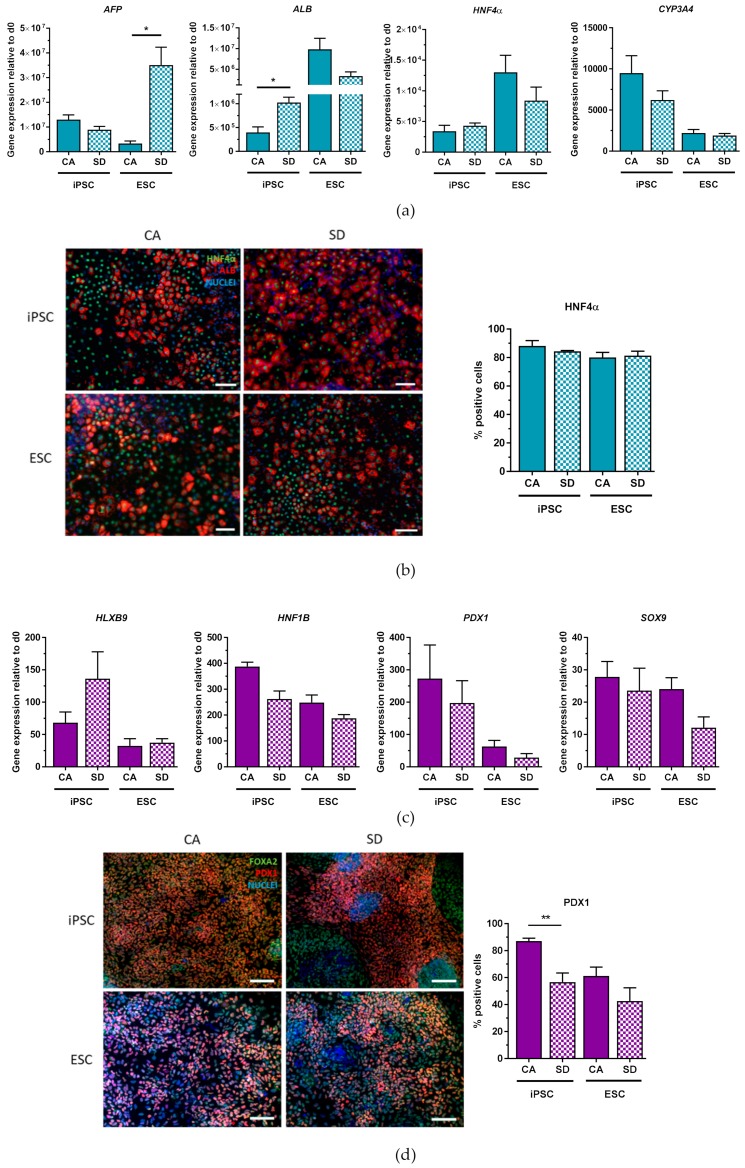
Hepatic and pancreatic differentiation potential of DE aggregates is confirmed by gene expression analysis and immunofluorescence staining. (**a**) qRT-PCR analysis of hepatic gene expression at day 14 of hepatic differentiation (n = 3). (**b**) Immunostaining of HNF4α (green) and ALB (red), nuclear stain DAPI (blue), and quantification of HNF4α+ cells at day 14 of hepatic differentiation (n = 3). (**c**) qRT-PCR analysis of pancreatic gene expression at day 10 of pancreatic differentiation (n = 3–4), (**d**) Immunostaining of PDX1 (red) and FOXA2 (green), nuclear stain DAPI (blue), and quantification of PDX1+ cells at day 10 of pancreatic differentiation (n = 3–5). Scale bars, 100 μm. Each value of gene expression was first normalized to the reference gene(s) and then to day 0 undifferentiated cells. Values are represented as the mean ± SEM. * *p* < 0.05, ** *p* < 0.01. All n values correspond to independent experiments.

To test lineage potential, DE aggregates were further differentiated towards hepatic-like, pancreatic, intestinal, and lung progenitor cells. Hepatic differentiation was performed using an established protocol [20]. Gene expression analysis on day 14 showed expression of key hepatic markers (Figure 2a) in both cell lines and conditions. Fetal-related hepatic marker alpha fetal protein (*AFP*) was expressed in hiPSC-derived hepatic-like cells in comparable amounts under both conditions. In contrast, the hESC-derived hepatic-like cells in the SD condition showed a significantly higher expression of *AFP* compared to the CA condition. The expression of the adult-related hepatic marker albumin (*ALB*) in the hiPSC-derived cells in the SD condition was significantly higher than those from the CA condition. Hepatic nucleus factor 4 alpha (*HNF4α*) expression in hiPSC-derived cells was similar, as was the expression in hESC-derived cells. The most abundant cytochrome P450 complex in the liver, *CYP3A4*, was expressed in comparable levels between the two conditions in both cell lines. In addition to qRT-PCR analysis, IF staining was performed and positive cells were counted to quantify lineage-specific marker gene expression for differentiated cells (day 7–day 14) as well as undifferentiated cultures (day 1) (Supplementary Materials Figure S3). IF staining revealed that both conditions and cell lines generated cells that were positive for the hepatic markers ALB and HNF4α (Figure 2b). HNFα quantification indicated that both conditions produced similar amounts of HN4α-expressing cells (80–88%). This data suggests that DE aggregates generated from both conditions can differentiate into hepatic-like cells that highly express key hepatic markers.

**Figure 3:**
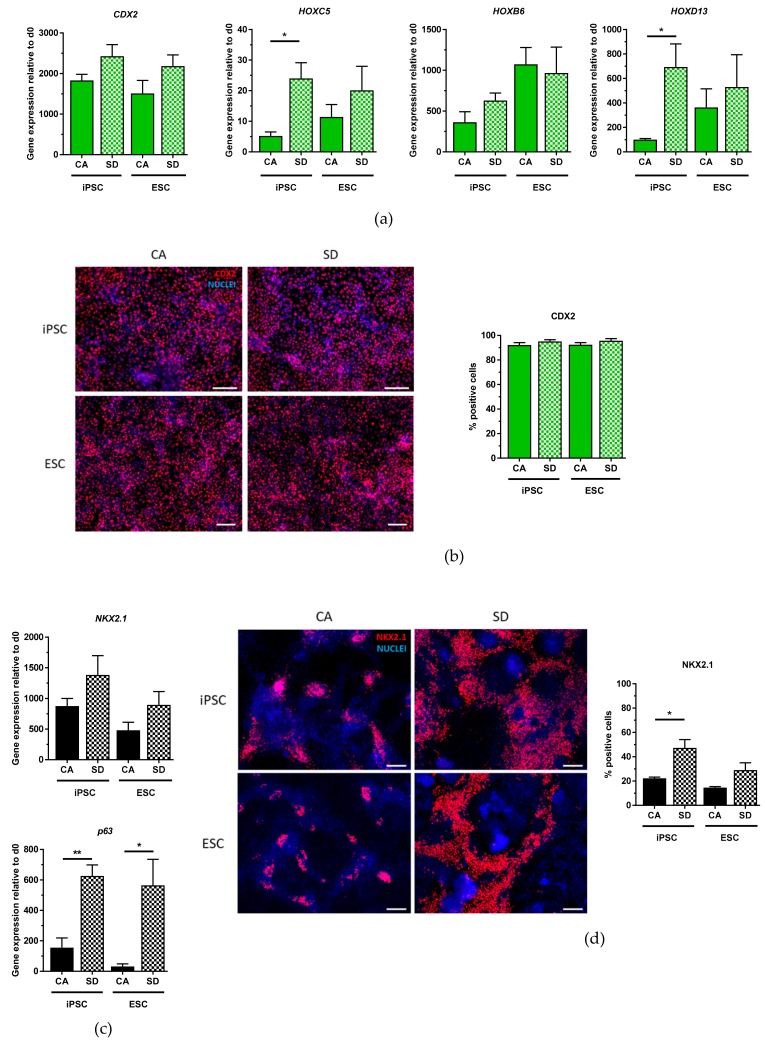
Intestinal and lung progenitor differentiation potential of DE aggregates is confirmed by gene expression analysis and immunofluorescent staining. (**a**) qRT-PCR analysis of *CDX2*, *HOXC5*, *HOXB6*, *HOXD13* expression at day 7 of intestinal differentiation (n = 3–4). (**b**) Immunostaining of CDX2 (red) nuclear stain DAPI (blue), and quantification of CDX2+ cells at day 7 of intestinal differentiation (n = 3–4). (**c**) qRT-PCR analysis of *NKX2.1* and *p63* expression between days 10–12 of lung differentiation (n = 3–4). (**d**) Immunostaining of NKX2.1 (red), nuclear stain DAPI (blue), and quantification of NKX2.1+ cells between days 10–12 of lung differentiation (n = 3). Scale bar, 100 μm. Each value of gene expression was first normalized to the reference gene(s) and then to day 0 undifferentiated cells Values are represented as the mean ± SEM. * *p* < 0.05. ** *p* < 0.01. All n values correspond to independent experiments.

Differentiation towards pancreatic cells was performed using a previously published protocol [21]. After further differentiating DE cells for 7 days, an increase in the gene expression in both cells lines and conditions of the pancreatic markers homeobox HB9 (*HLXB9*), hepatocyte nuclear factor 1 homeobox B (*HNF1B*), pancreatic and duodenal homeobox 1 (*PDX1*), and *SOX9* could be detected (Figure 2c). To confirm these findings, differentiated cells were also co-stained for FOXA2, an important hallmark of endoderm and pancreatic progeny, and PDX1 (Figure 2d). FOXA2 protein was almost homogeneously expressed with very few FOXA2- areas in both conditions and cell lines. PDX1+ cells were more abundant in the CA condition (87%) compared to the SD condition (57%) for hiPSC. The same pattern was seen for the hESC-derived cells, with 61% PDX1+ cells from the CA and 43% PDX1+ cells from the SD condition (Figure 3d). Thus, these results indicate that DE aggregates can also be differentiated towards pancreatic precursor cells. The next step was to test whether DE aggregates could also generate intestinal and lung-progenitor cells.

By adapting a protocol from Spence et al., dissociated aggregates were differentiated into intestinal cells for an additional four days [22]. By day 7 of differentiation, both hiPSC and hESC derivatives in both conditions expressed the critical hindgut marker caudal type homeobox 2 (*CDX2*) (Figure 3a) in combination with *HOXC5*, *HOXB6*, and *HOXD13*, homeobox transcription factor genes important for posterior gut patterning [31]. iPSCs derived from the SD condition had significantly higher *HOXC5* and *HOXD13* expression compared to those from the CA condition. The overall higher expression of *HOXB6* and *HOXD13* and lower expression of *HOXC5* suggests that these cells are more hindgut intestinal cells than midgut. Immunofluorescence staining showed a homogenously positive population of CDX2+ cells (Figure 3b). Upon quantification, between 92–96% of the cells were positive for CDX2, with no significant differences between CA and SD conditions.

In addition to intestinal differentiation, hPSCs were also differentiated towards lung progenitor cells using a protocol adapted from Huang et al. [32]. Initial differentiation of cells from the CA condition yielded very few lung progenitor cells according to NK2 homeobox 1 (*NKX2.1*) expression, the earliest known marker for lung cells (data not shown). Introduction of a passing step on day 7 led to an increase of *NKX2.1*-expressing cells. After optimizing the differentiation protocol, DE cells from SD, as well as CA conditions, were capable of differentiating into lung progenitor cells shown by expression of *NKX2.1* and the basal cell marker *p63* (Figure 3c) on days 10–12 of differentiation, with cells from the SD condition expressing significantly higher levels of *p63* than cells from the CA condition. Visual differences between the two conditions were observed upon NKX2.1 IF staining (Figure 3d). Specifically, cells from the CA condition tended to form small clusters of NKX2.1+ cells whereas NKX2.1+ cells from the SD condition tended to be more spread out throughout the cell culture plate (Figure 3d). Quantification of NKX2.1+ cells showed that all conditions were able to give rise to lung progenitor cells. However, hiPSC-derived cells from the SD condition produced a significantly greater number of NKX2.1+ cells (47%) compared to the hiPSC CA condition (22%) (Figure 3d). Similarly, 29% of hESC-derived cells from the SD condition were positive for NKX2.1, compared to 15% of hESC-derived cells from the CA condition (Figure 3d). Putting all these results together, IF staining of differentiated DE aggregates corroborate qRT-PCR analysis and show that DE aggregates from both conditions can differentiate into hepatic-like, pancreatic, intestinal, and lung progenitor cells.

### 3.3. DE Differentiation in a Fully-Controllable, Stirred-Tank Bioreactor

**Figure 4:**
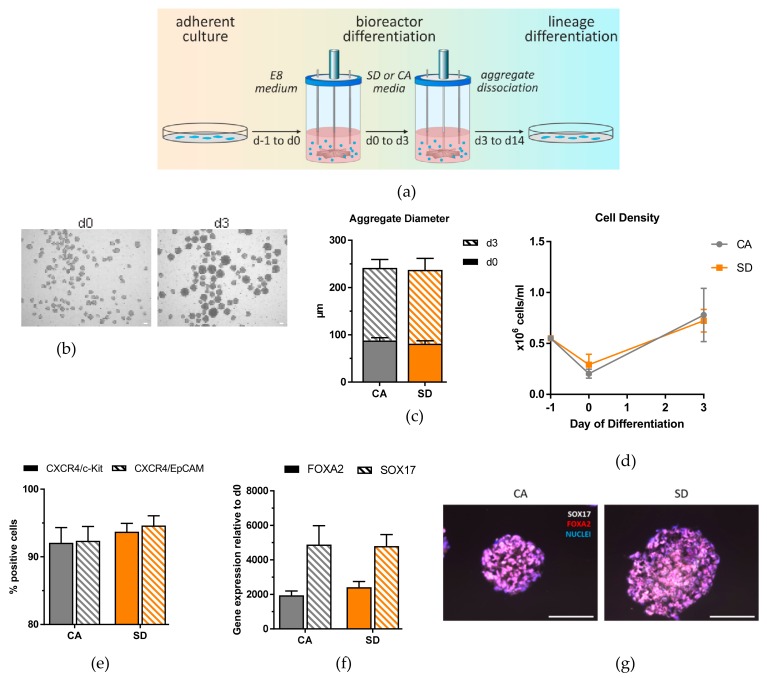

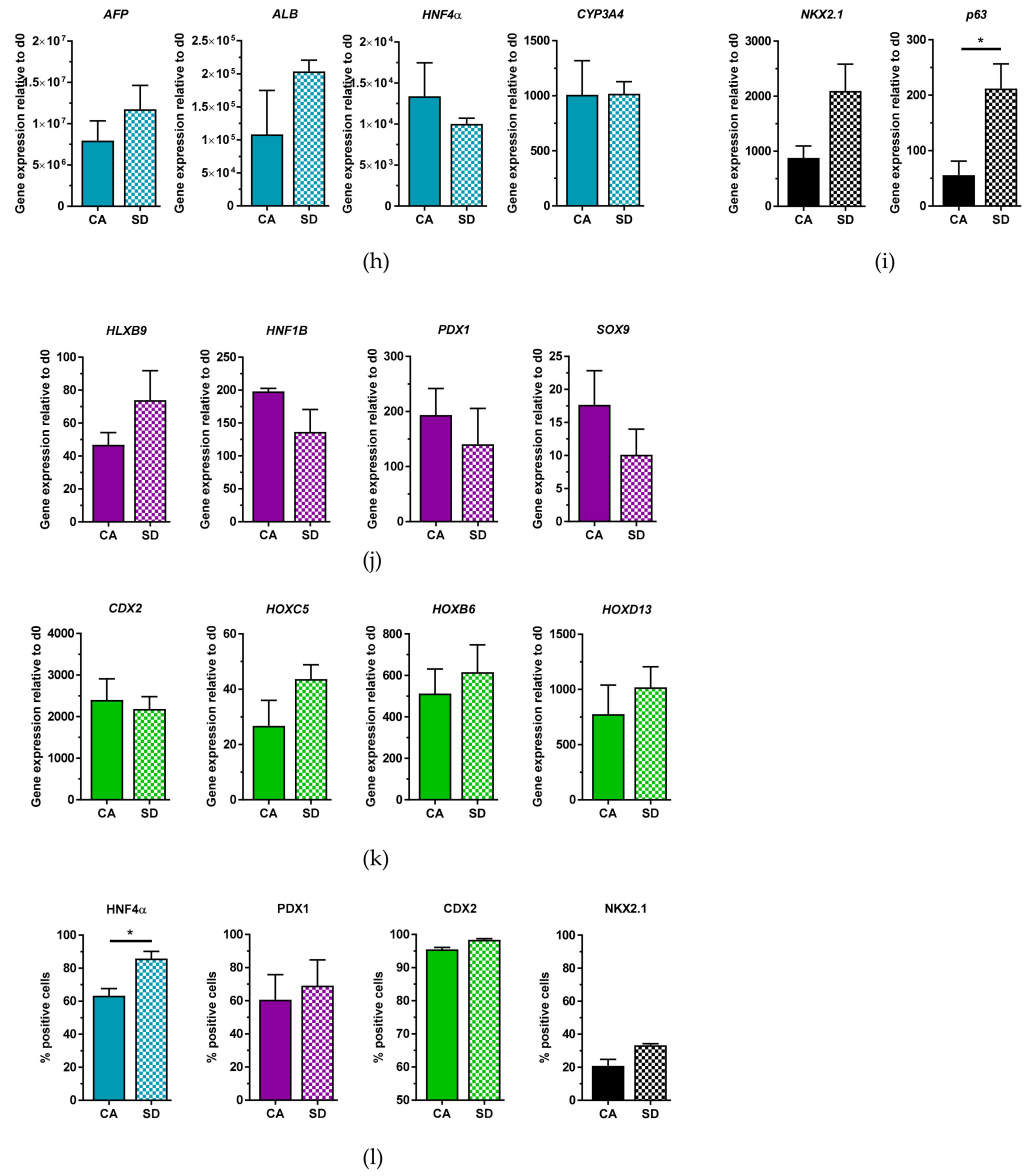
iPSC DE differentiation in suspension is scalable to a bioreactor and can give rise to multiple lineages upon further differentiation. (**a**) Schematic overview of DE differentiation in bioreactor. (**b**) Representative brightfield images at day 0 and day 3 of differentiation. (**c**) Diameter measurements of aggregates at day 0 and day 3 of differentiation. (**d**) Cell density measurements at day 0 and day 3 of differentiation. (**e**) DE differentiation efficiency measured by flow cytometry analysis. (**f**) qRT-PCR analysis of *FOXA2* and *SOX17* at day 3 of differentiation. (**g**) Immunostaining of FOXA2 (red) and SOX17 (white), nuclear stain DAPI (blue), of sectioned aggregates at day 3 of differentiation. (**h**) qRT-PCR analysis at day 14 of hepatic differentiation. (**i**) qRT-PCR analysis of *NKX2.1* and *p63* expression at day 10 of lung differentiation. (**j**) qRT-PCR analysis of pancreatic gene expression at day 10 of pancreatic differentiation. (**k**) qRT-PCR analysis of *CDX2*, *HOXC5*, *HOXB6*, and *HOXD13* expression at day 7 of intestinal differentiation. (**l**) Quantification of HNFα+, PDX1+, CDX2+, and NKX2.1+ cells from immunostainings, respectively. Each value of gene expression was first normalized to the reference gene(s) and then to day 0 undifferentiated cells. Values are represented as the mean ± SEM. * *p* < 0.05. All data represent three independent experiments. Scale bar, 100 μm.

To test further scalability of the DE differentiation protocol established in the Erlenmeyer flask, a 150 mL fully controllable, stirred bioreactor was used to differentiate the hiPSC line towards DE (Figure 4a). The protocol did not need to be adapted or modified to generate aggregates at day 0 (Figure 4b) and to successfully differentiate the aggregates into DE cells, reflecting the robustness of the established method. Aggregates generated in the bioreactor were on average smaller, 88.2 µm for the CA condition, and 81.4 µm for the SD condition, than those generated in the Erlenmeyer flask (Figure 4c). These aggregates followed a similar growth pattern as those in the Erlenmeyer flask, with a cell density of 7.80 × 10^5^ cells/mL for the CA condition and 7.23 × 10^5^ cells/mL for the SD condition by day 3 of differentiation (Figure 4d). In total, an average of 1.12 × 10^8^ DE-cells could be generated in one 150 mL bioreactor. Parameters such as dissolved oxygen (DO) and pH were also monitored during the three days of DE differentiation. DO concentration was different between the two conditions (Supplementary Materials Figure S2a). A spike in DO concentration occurred with every medium exchange, and by day 3 of differentiation, the dissolved oxygen concentration in the SD condition decreased only slightly from 100% to 96%, while in the CA condition, the dissolved oxygen concentration decreased to 38%. The pH of both conditions followed a similar pattern during differentiation, with a spike in pH occurring with every daily medium change. By day 3, the pH slowly decreased to 6.72 and 6.58 in the CA and SD condition, respectively (Supplementary Materials Figure S2b). Flow cytometric analysis of aggregates at day 3 revealed that 92–95% of cells from both conditions were positive for the DE marker combinations CXCR4/c-Kit and CXCR4/EpCAM (Figure 4e). Gene expression analysis and IF staining also confirmed the expression of SOX17 and FOXA2 with homogenous distribution of both markers throughout the aggregates (Figure 4f,g). These results show that DE differentiation in the bioreactor produces a large quantity of DE cells with similar efficiencies as in the Erlenmeyer flask.

After successful DE generation in the bioreactor, we tested the multi-lineage capability of these cells. After hepatic differentiation, qRT-PCR analysis of differentiated DE aggregates showed generation of hepatic-like cells (Figure 4h), pancreatic cells (Figure 4j), intestinal cells (Figure 4k), as well as lung progenitor cells (Figure 4i). These results were confirmed on the protein level by quantification of positive cells after IF staining (Figure 4l). HNF4α quantification of cells differentiated towards a hepatic lineage showed that the CA condition produced fewer positive cells in the bioreactor (63%) compared to the Erlenmeyer flask (88%), while the SD condition produced similar amounts of cells in the bioreactor (86%) compared to the Erlenmeyer flask (84%). By day 10 of pancreatic differentiation, a similar pattern was observed where the CA condition in the bioreactor showed fewer PDX1+ cells (61%) compared to the Erlenmeyer flask (87%) while the SD condition had comparable efficiencies (69%). Aggregates generated in the bioreactor were also capable of generating CDX2+ cells by day 7 of intestinal differentiation, with 96% of cells from the CA condition and 98% of cells from the SD condition positive for CDX2. These percentages were comparable to the Erlenmeyer flask (92–95%). Lung progenitor differentiation of aggregates from the bioreactor was similar to that in the Erlenmeyer flask, with 21% NKX2.1+ cells from the CA condition and 33% NKX2.1+ cells from the SD. Overall, aggregates generated from the bioreactor are capable of differentiating not only towards DE with high efficiency but also further differentiate into hepatic-like, pancreatic, intestinal, and lung-progenitor cells with similar efficiencies as DE aggregates generated from the Erlenmeyer flask.

### 3.4. Dissociated DE Aggregates Can Be Cryopreserved and Retain Multi-Lineage Differentiation Potential

**Figure 5:**
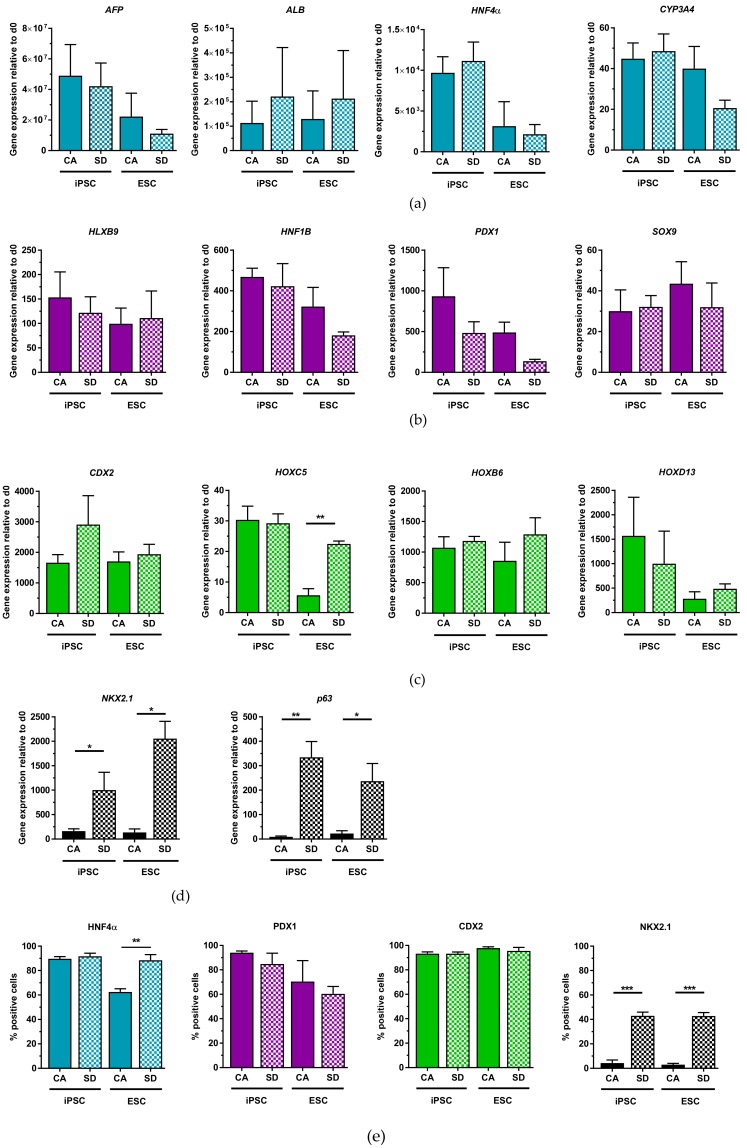
Cryopreservation does not compromise all multi-lineage capability of DE aggregates. (**a**) qRT-PCR analysis of hepatic gene expression of thawed cells at day 14 of hepatic differentiation (n = 3). (**b**) qRT-PCR analysis of pancreatic gene expression of thawed cells at day 10 of pancreatic differentiation (n = 3–5). (**c**) qRT-PCR analysis of *CDX2* expression of thawed cells at day 7 of intestinal differentiation (n = 3). (**d**) qRT-PCR analysis of *NKX2.1* and *p63* expression of thawed cells between days 10–13 of lung differentiation (n = 3–4). (**e**) Quantification of HNFα+ cells (n = 3), PDX1+ cells (n = 3–4), CDX2+ cells (n = 3), and NKX2.1+ cells (n = 3) from immunostaining, respectively. Each value of gene expression was first normalized to the reference gene(s) and then to day 0 undifferentiated cells. Values are represented as the mean ± SEM. * *p* < 0.05, ** *p* < 0.01, *** *p* < 0.001. All n values correspond to independent experiments.

Cryopreservation of DE cells allows for uncoupling of DE production and subsequent differentiation and enables production of the required DE lineages off-the-shelf. Thus, we investigated whether DE aggregates produced in Erlenmeyer flasks could be cryopreserved and maintain their multi-lineage potential upon thawing. qRT-PCR analysis of each differentiated lineage generated from thawed DE cells showed generation of markers for hepatic-like (Figure 5a), pancreatic (Figure 5b), intestinal (Figure 5c), as well as lung progenitor cells (Figure 5d) from SD and CA conditions. Specifically, for hepatic differentiation, cryopreserved hiPSC-derived DE cells had higher *AFP* expression compared to non-cryopreserved aggregates, while hESC-derived cells had comparable levels of *AFP* in both conditions. *ALB* expression was also comparable between both cell lines and conditions. The detoxification enzyme *CYP3A4* showed a much lower expression level in the cryopreserved cells than in the non-cryopreserved cells. Although there are slight differences in the gene expression pattern of thawed DE cells compared to their non-cryopreserved counterpart, thawed DE cells differentiated towards the hepatic lineage express key hepatic markers. After 7 days of pancreatic differentiation, cryopreserved cells showed an increase in gene expression of *HNF1B*, *HLXB9*, *PDX1*, and *SOX9* (Figure 5b) compared to day 0 undifferentiated cells, which is comparable to results from the Erlenmeyer flask. Interestingly, *HOXC5* and *HOXD13* gene expression analysis after intestinal differentiation showed that thawed iPSC-derived DE cells from the CA condition expressed much higher levels of *HOXC5* and *HOXD13* compared to non-cryopreserved cells (Figure 5c). For lung differentiation, thawed DE cells showed comparable expression of *NKX2.1* and *p63* to non-cryopreserved cells except the CA condition, which had lower *NKX2.1* and *p63* expression compared to non-cryopreserved DE cells that were differentiated towards lung (Figure 5d). Taking all these results together, cryopreserved DE cells maintain multi-lineage potential and are capable of differentiating towards hepatic-like, pancreatic, intestinal, and lung progenitor cells.

In addition to qPCR analysis, IF staining was also performed to quantify differentiation efficiency into the different lineages of the DE (Figure 5e). IF staining and quantification of HNF4α cells after 14 days of hepatic differentiation revealed that only the hESC-derived cells from the CA condition had a lower expression (63%), while the other conditions had similar expression to non-cryopreserved cells. Immunofluorescent quantification of PDX1+ cells after pancreatic differentiation showed slightly higher expression (60–94%) compared to non-cryopreserved DE cells (42–87%) (Figure 5e). Cryopreserved cells differentiated towards the intestinal lineage resulted in 93–98% of CDX2+ cells in all conditions by day 7 of differentiation (Figure 5e), which is similar to the differentiation efficiency of non-cryopreserved DE cells (92–96%). After lung progenitor differentiation, cells from the SD condition were able to produce similar amounts of NKX2.1 expressing cells (43%) compared to their non-cryopreserved counterpart (29–47%) (Figure 5e). However, thawed DE-cells from the CA condition produced very few NKX2.1-expressing cells (Figure 5e). Only 4% of hiPSC-derived cells from the CA condition and 3% of hESC-derived cells from the CA condition showed NKX2.1 expression. Results of the IF quantification confirmed qRT-PCR results and demonstrated that cryopreserved DE cells maintain their multi-lineage potential when thawed and differentiated into the different DE lineages.

## 4. Discussion

In the present study, we report the development of a versatile, xeno-free, and scalable protocol for the generation of multipotent DE from hPSCs using industry-scale bioreactors. We also provide evidence that cells can be cryopreserved at the DE stage for later off-the-shelf differentiation and retain their multi-lineage potential.

In the past, one impeding obstacle which prevented the use of dynamic cultures for hPSC applications was hPSC sensitivity for enzymatic dissociation and failure to form proliferating aggregates or single cell-derived clones. This was overcome by the advent of the small molecule Rho kinase inhibitor Y-27632 [33]. We have shown that with this molecule, undifferentiated hPSC form floating aggregates and can be expanded under defined conditions over several passages while maintaining their three-lineage differentiation potential [34]. As a logical continuation of our research, we have now addressed the questions as to whether undifferentiated hPSC 3D aggregates can be directly differentiated into DE and its derivatives in dynamic suspension, if industrial scale bioreactor culture is possible, and whether multi-lineage differentiation potential is maintained after cryopreservation at the DE stage.

For proof-of-principle, we used Erlenmeyer flasks stirred by an orbital shaker as the main suspension culture system. This system has been well-established, and can be affordably transferred to any laboratory [35]. Suspension aggregate formation was performed in E8 medium plus Y-27632, representing a defined condition for the culture of undifferentiated hPSCs. This procedure eliminates the need for xeno-matrix substances, like Matrigel^®^, which are commonly used as substrates for cell attachment in 2D. Even though both hiPSCs and hESCs were capable of generating stable floating aggregates, the HES3 line was more sensitive to dissociation and aggregate formation during the first 24 h of suspension culture. This suggests that it is important to optimize certain parameters such as cell inoculation density based on the cell line being used. Further optimization in the direction of protecting singularized cells from apoptosis during aggregate formation in dynamic suspension would provide the opportunity to include cell lines that are delicate in this regard, but this was not further addressed in this study.

Characterization of DE aggregates produced in the Erlenmeyer flask revealed that we obtained highly homogeneous DE populations with greater than 92% of cells positive for CXCR4/c-Kit, CXCR4/EpCAM under all conditions for all three cell lines. In addition, the DE transcription factors *SOX17* and *FOXA2* were highly expressed. This is a clear improvement in differentiation efficiencies compared to other suspension protocols [14,17], and was an ideal starting point to efficiently derive different downstream lineages. The growth rate of the two conditions and total number of cells retrieved after differentiation were significantly higher than those from 2D adherent cultures, which supports the use of suspension culture differentiation over 2D adherent culture for generating large quantities of DE cells.

Dissociated DE aggregates generated in Erlenmeyer flasks were capable of differentiating into the different lineages of the DE with similar efficiencies as to what has been published. In general, the HES3 line was slightly less efficient during differentiation. We have not further investigated this observation, but the lower differentiation efficiency may be due to a higher vulnerability of this cell line to the forces acting on the aggregates under dynamic culture conditions. The efficiency of hepatic differentiation achieved was relatively high with 84–88% of cells that were positive for HNF4α, which is an essential transcription factor in the transition of endoderm towards hepatic fate [36]. This efficiency is even better than results others recently achieved with defined, small molecule-based hepatic differentiation protocol (72% AFP+HN4A+ cells, [37], 60% ALB, [13]). Pancreatic lineage potential of our DE aggregates was demonstrated by differentiation towards PDX1+ cells. hiPSC-derived cells from the CA condition differentiated with similar efficiencies (87%) compared to what Yabe et al. reported (92.1%). Intestinal lineage differentiation was highly efficient, with 92–95% of cells expressing CDX2 by day 7 of differentiation. Lung progenitor differentiation of DE aggregates revealed that the SD condition led to significantly more NKX2.1+ cells compared to the CA condition. The reason for this was not addressed in further detail, but it indicates that although our characterization with standard DE markers (CXCR4, c-Kit, EpCAM, FOXA2, and SOX17) on day 3 led to similar and homogeneous results between the two conditions, the DE aggregates generated with the SD condition can more efficiently differentiate towards lung progenitor cells using our lung differentiation protocol. Differentiation towards lung progenitor cells from hiPSCs using the SD condition is comparable to another established adherent lung differentiation protocol which reported up to 40% NKX2.1+ cells using the SD kit [30]. This comparison suggests that our method for DE generation is a competitive alternative to other methods of DE generation not only because of good differentiation efficiency but also because there is the added advantage that only one DE protocol is needed to differentiate towards many lineages of the DE.

After testing the feasibility of suspension culture DE differentiation in Erlenmeyer flasks, the protocol was adapted to a stirred-tank bioreactor, which has been previously shown to maintain hPSCs in suspension [7] and can be used to differentiate them to cardiomyocytes [9], endothelial cells [11], and macrophages [38]. Interestingly, no further optimization to the media composition was necessary to generate DE in the bioreactor. An average of 1.12 × 10^8^ iPSC-derived DE cells could be generated in one 150 mL bioreactor, which equates to 7.5 × 10^5^ cells/mL, and is slightly lower than cell numbers achieved in our Erlenmeyer flasks differentiation experiments (1.2 × 10^6^ cells/mL). Generated cell numbers in the bioreactor are comparable to what was reported so far. Lock et al. reported 4–5 × 10^5^ cells/mL and Shinohara et al. reported 5–8 × 10^5^ cell/mL using their suspension culture system [15,16]. With our current system, a 2 L bioreactor could provide a sufficient amount of DE cells for human islet differentiation and transplantation [1], while upscaling to 19 L bioreactor could provide a sufficient amount of DE cells that could be differentiated into hepatic cells to treat a 70 kg patient [2]. The differences in aggregate diameter, cell density, and marker gene expression between Erlenmeyer flask and bioreactor could be attributed to the physical differences between each platform including shape of vessel, method of stirring, and shear stress as a result of both those factors. Despite these differences, this bioreactor setup is suitable to efficiently generate multipotent DE. There may be further potential for optimizing parameters like stirring speed and impeller design and for harmonizing pH control, dissolved oxygen, and feeding to generate a further increase in cell density and reduce the volume of media needed to attain clinically relevant cell numbers. These variables have to be addressed in future work. With our current bioreactor system and protocol, we can successfully generate large quantities of DE cells that can be used for multi-lineage differentiation that can potentially be used for cell therapy, drug screening, and disease-modelling purposes.

The last point we addressed was the ability to cryopreserve dissociated DE aggregates generated in the Erlenmeyer flask. Cryopreservation of DE cells allows for the storage of DE cells for future experiments, it reduces costs as one large stock of cells can be created, reducing the number of differentiations performed, and it can improve reproducibility when performing experiments. In addition, cryopreservation could separate the steps of mass cell production, cell-type-specific differentiation and final therapeutic or scientific destination. For these reasons, we sought to cryopreserve our DE cells and test whether they maintain their multi-lineage potential upon thawing. Our results show that cryopreserved DE cells from the CA and SD conditions were capable of differentiating towards hepatic-like, pancreatic, and intestinal cells in general with similar efficiency as non-cryopreserved DE cells. However, in the case of hepatic differentiation, expression levels of markers for more mature hepatic cells like *CYP3A4* and *ALB* are lower than after differentiation of non-cryopreserved cells, suggesting a delay in differentiation or even a loss of a specific progenitor subpopulation due to cryopreservation. Differences in the thawed iPSC-derived DE cells from the CA condition were also observed during intestinal differentiation. These cells expressed higher levels of *HOXC5* and *HOXD13* compared to non-cryopreserved cells, suggesting a stimulating effect of cryopreservation that resulted in better differentiation. However, the same pattern of higher expression of *HOXB6* and *HOXD13* compared to *HOXC5* can be observed in the Erlenmeyer flask, the bioreactor, and upon thawing, which suggests that these cells retain their hindgut phenotype even after cryopreservation [31]. While lung differentiation of thawed DE cells from the SD condition was as efficient as non-cryopreserved cells, differentiation of thawed cells from the CA condition was less efficient. One possible reason for this is that the differentiation protocol and media must be further optimized to support higher *NKX2.1* expression of cells from the CA condition. Seeding density of thawed cells may also need to be further optimized, as we have seen that this greatly affects differentiation efficiency. Furthermore, DE cells primed for anterior foregut differentiation may be more sensitive to cryopreservation. Despite the lower lung progenitor differentiation efficiency in the CA condition and a possible delayed maturation of hepatic cells, DE cells from both SD and CA conditions maintain their multi-lineage potential upon cryopreservation, making the process for DE generation and differentiation more flexible.

Taken together, our study provides a versatile, robust, and highly efficient protocol for the generation of DE in dynamic suspension cultures with industrial/clinical scale potential and enables off-the-shelf supply of DE cells for production of different DE lineages. The described protocols represent a major step towards the routine application of cellular therapies, toxicity screening, and disease modeling with endoderm cell types derived from hPSCs.

## Supplementary Materials

The following are available online at https://www.mdpi.com/2073-4409/8/12/1571/s1, Figure S1: Generation and characterization of DE in Erlenmeyer flasks utilizing a third cell line (iPSC). Figure S2: Dissolved oxygen and pH monitoring during DE differentiation in bioreactor. Figure S3: DE and lineage marker stains on undifferentiated iPSCs and ESCs grown as monolayers. Table S1: Primary Antibody List. Table S2: Secondary Antibody List. Table S3: Primer Sequences.
