# Supplementary material for: Chemically-Defined, Xeno-Free, Scalable Production of hPSC-Derived Definitive Endoderm Aggregates with Multi-Lineage Differentiation Potential
Source: Cells. 2019 Dec 4;8(12):1571. doi: 10.3390/cells8121571 (PMC6953099; doi:10.3390/cells8121571)
Supplement: Supplementary file 1 [file cells-08-01571-s001.pdf]

Supplementary Materials:

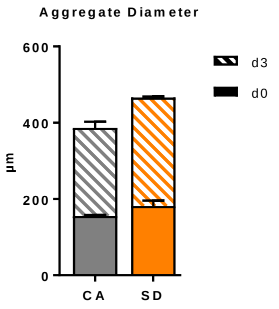

(a)

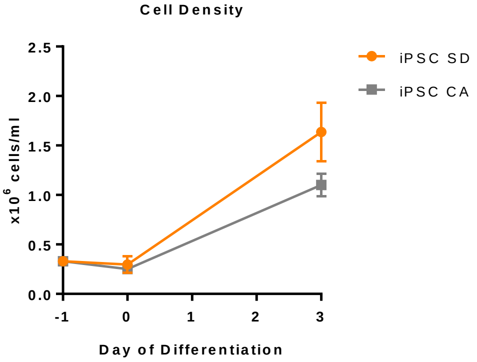

(b)

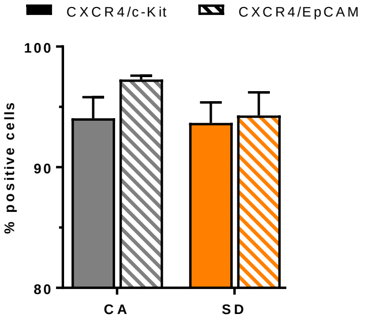

(c)

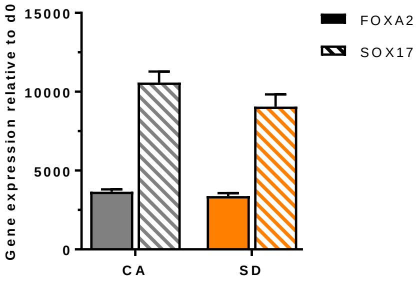

(d)

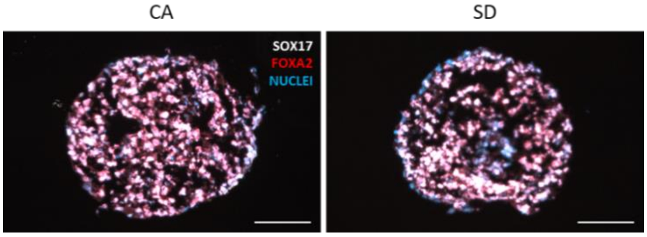

(e)

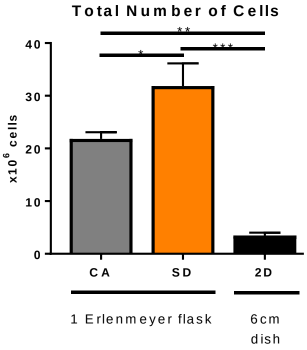

(f)

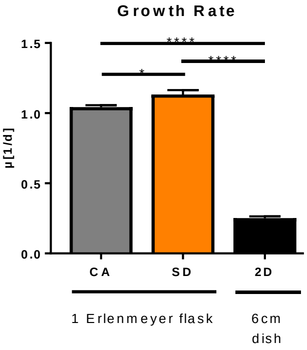

(g)

Supplemental Figure 1. Generation and characterization of DE in Erlenmeyer flasks utilizing a third cell line (iPSC). (a) Diameter measurement of aggregates at d0 and d3 of differentiation. (b) Cell density measurements at d0 and d3 of differentiation. (c) Definitive endoderm efficiency quantification based on flow cytometry analysis of CXCR4/c-Kit and CXCR4/EpCAM double positive cells at d3 of differentiation (n = 3). (d) qRT-PCR analysis of FOXA2 and SOX17 expression at d3 of differentiation (n = 3). (e) Immunostaining of FOXA2 (red) and SOX17 (white) of DE aggregates at d3 of differentiation (n = 3), nuclear stain DAPI (blue). (f)

Total number of DE cells after differentiation compared to adherent 2D culture (n = 3-5). (g) Growth rate of CA and SD conditions from d0 to d3 of differentiation compared to adherent 2D culture (n = 3-5). Scale bars, 100 µm. Each value of gene expression was first normalized to the reference gene(s) and then to d0 undifferentiated cells. Values are represented as the mean ± SEM. *P<.05, **P<0.01, ****P<0.0001. All n values correspond to independent experiments.

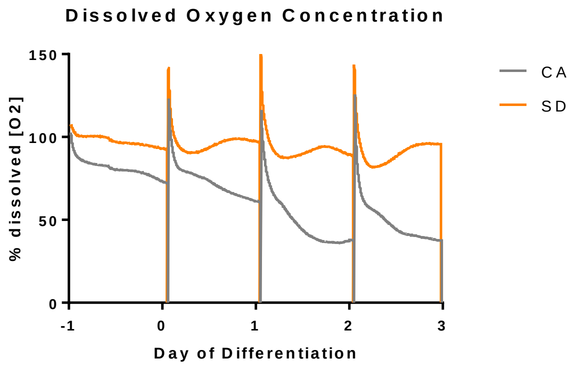

(a)

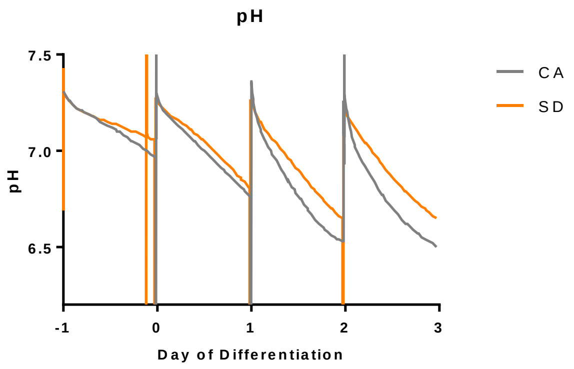

(b)

Supplemental Figure 2. Dissolved oxygen and pH monitoring during DE differentiation in bioreactor. (a) Representative time course measurement of dissolved oxygen concentration of bioreactor in the CA and SD conditions during DE differentiation. (b) Representative pH measurement of the CA and SD condition during DE differentiation

Supplemental Figure 3

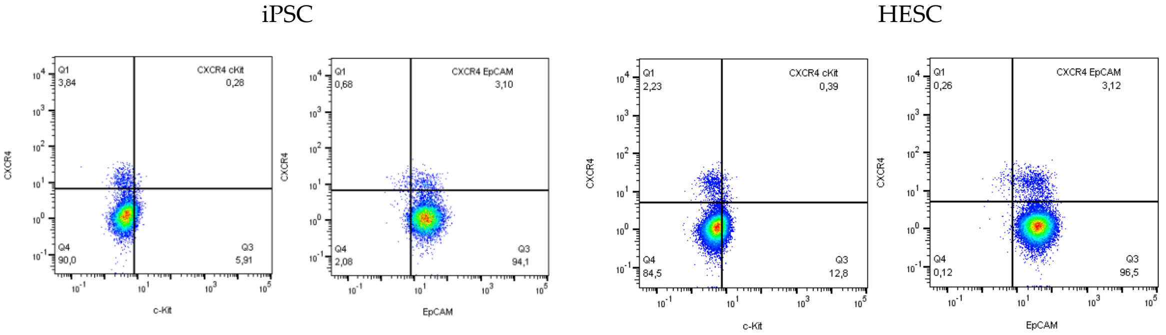

(a)

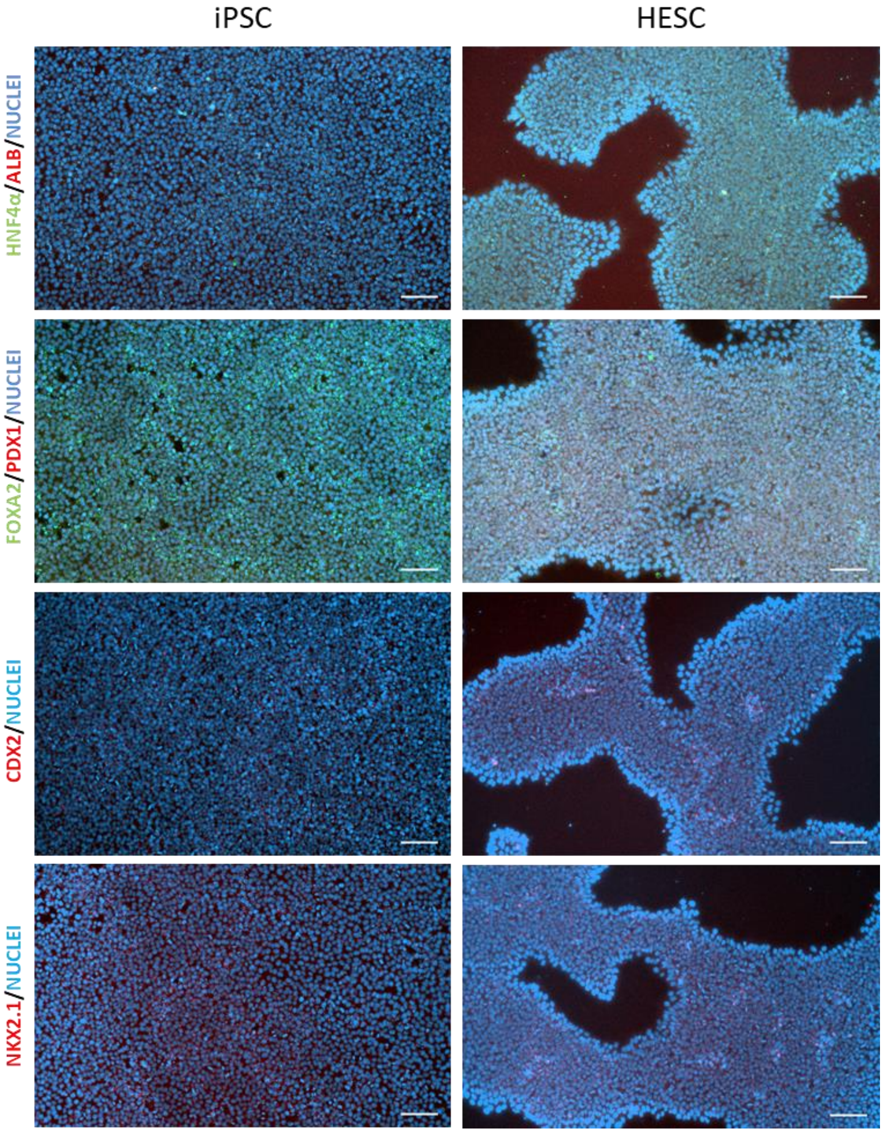

(b)

Supplemental Figure 3. DE and lineage marker stains on undifferentiated iPSCs and ESCs grown as monolayers. (a) Flow cytometry analysis of CXCR4/c-Kit and CXCR4/EpCAM on undifferentiated cells. (b) Lineage marker stains on undifferentiated cells. Scale bars, 100 μm.

Supplemental Table 1. Primary Antibody List.

Primary Antibody	Lineage	Company	Catalog No.	Dilution
Human SOX17	DE	R&D Systems	AF1924	1:200
Anti-HNF3β/FOXA2	DE, pancreatic	Merck Millipore	07-633	1:200
ALBUMIN	Hepatic	Bethyl	A80-229A #25	1:1000
HNF4α	Hepatic	Santa Cruz	Sc-8987	1:400
PDX1	Pancreatic	R&D Systems	#AF2419	1:300
CDX2	Intestinal	BioGenex	MU392A-5UC	1:100
TTF-1 (NKX2.1)	Lung Progenitor	Life Technologies	180221	1:100

Supplemental Table 2. Secondary Antibody List.

Secondary Antibody	Lineage	Company	Catalog No.	Dilution
Donkey anti-goat cy3	DE	Jackson Immunoresearch	705-105-147	1:200
Donkey anti-rabbit AF647	DE	Jackson Immunoresearch	711-606-647	1:200
Goat anti-rabbit cy3	Hepatic	Jackson Immunoresearch	171-165-152	1:1000
Donkey anti-goat A488	Hepatic	Jackson Immunoresearch	705-545-003	1:400
Donkey anti-rabbit AF488	Pancreatic	Jackson Immunoresearch	711-545-152	1:300
Donkey anti-goat AF647	Intestinal, Pancreatic	Jackson Immunoresearch	705-605-147	1:100
Donkey anti-mouse cy3	Lung Progenitor	Jackson Immunoresearch	715-165-150	1:100

Supplemental Table 3. Primer Sequences.

Primer Name	Primer Sequence
AFP	Assay # HS01040595_G1
ALB	Assay # Hs01040595_g1
CDX2 Fw	5′-GGCAGCCAAGTGAAAACCAG-3′
CDX2 Rev	5′-TCCTTTGCTCTGCGGTTCTG-3′
CK7	Assay # Hs 00559840_m1
CK19	Assay # Hs00761767_s1
CYP3A4	Assay # Hs_00604506_m1
EEF1A1 Fw	5′-CATCAAAGCAGTGGACAAGAAG-3′
EEF1A1 Rev	5′-GGGTGGCAGGTATTAGGGATAA-3′
FOXA2 Fw	5′-GGGAGCGGTGAAGATGGA-3′
FOXA2 Rev	5′-TCATGTTGCTCACGGAGGAGTA-3′

G6PD Fw	5′-AGGCCGTCACCAAGAACATTCA-3′
G6PD Rev	5′-CGATGATGCGGTTCCAGCCTAT-3′
GAPDH	Assay # HS00171403_M1
HLXB9 Fw	5′-TCCACCGCGGGCATGATC-3′
HLXB9 Rev	5′-GCTTGGGCCGCGACAGGTA-3′
HNF1B Fw	5′-GAGGAATGCAACAGGGCAGAATG-3′
HNF1B Rev	5′-GAATGCCTCCTCCTTCCTGCG-3′
HNF4	Assay # Hs00604438_m1
NKX2.1 Fw	5′-CGGCATGAACATGAGCGGCAT-3′
NKX2.1 Rev	5′-GCCGACAGGTTCTGTTGCTTG-3′
PDX1 Fw	5′-CGTCCAGCTGCCTTTCCCAT-3′
PDX1 Rev	5′-CCGTGAGATGTACTTGTTGAATAGGA-3′
RPLP0 Fw	5′-ACGGATTACACCTTCCCACTT-3′
RPLP0 Rev	5′-TCTTCCTTGGCTTCAACCTTAG-3′
SOX17 Fw	5′-CCAAGGGCGAGTCCCGTATC-3′
SOX17 Rev	5′-CACGACTTGCCCAGCATCTTG-3′
SOX9 Fw	5′-GCGGAGGAAGTCGGTGAAGAACG-3′
SOX9 Rev	5′-CTGGGATTGCCCCGAGTGCTC-3′
TBP Fw	CAACAGCCTGCCACCTTACGCTC-3′
TBP Rev	AGGCTGTGGGGTCAGTCCAGTG-3′
TTR	Assay # Hs00174914_m1
TUB1A1 Fw	GGCAGTGTTTGTAGACTTGGAACCC-3′
TUB1A1 Rev	TGTGATAAGTTGCTCAGGGTGGAAG-3′

IF staining for Hepatic Differentiation

Cells were washed three times with phosphate-buffered saline (PBS), fixed in 4% formaldehyde for 20 min at 4 °C, washed three times with PBS and permeabilized with 0.2% Tween 20 (Roth #9127.1) plus 0.1% IGEPAL® CA-630 (Sigma #I8896) in Tris-buffered saline (TBS) for 20 min. The permeabilized cells were washed three times with TBS, blocked with 3% BSA in TBS for 1 h, and washed once with TBS. Then, the cells were incubated with primary antibodies (Supplemental Figure 1) in TBS containing 1% BSA over night at 4°C. The next day cells were washed three times with TBS and incubated with secondary antibodies (Supplemental Figure 2) diluted in TBS 1h at RT. Cells were washed three times with TBS and counterstained with DAPI (Invitrogen #D1306). Staining was analyzed using an Olympus IX71 microscope equipped with appropriate filters for fluorescence detection, and images were processed using the cellSens software (Olympus).

IF staining for Pancreatic Differentiation

Differentiated cells were fixed for 10-20 min with 4% paraformaldehyde. Cell culture slides were washed with PBS and subsequently blocked for 20 min in PBS plus 0.2% Triton X-100 and 1 mg/ml $NaBH_4$ and 5% donkey serum (Dianova #017-000-121). Primary and secondary antibodies (Supplemental Figure 1 and 2) were diluted in PBS with 0.1% Triton X-100 plus 0.1% donkey serum. Primary antibodies were incubated overnight at 4°C. Secondary antibodies were incubated for 1 h at room temperature. Nuclei were stained with DAPI. Images of pancreatic differentiation were taken using the Olympus IX81 microscope. Image processing was performed with cellSens software (Olympus).

IF Staining for Intestinal and Lung Differentiation

Cells were fixed with 4% PFA for 20 minutes and washed 3 times with PBS. The cells were incubated in blocking-permeabilization solution (TBS, 5% donkey serum, 0.025% Triton-X 100) for 20 minutes and then washed 3 times with PBS. Primary antibody (Supplemental Figure 1) was added for 1 hour at RT. The cells were washed 3 times with PBS, and incubated with secondary antibody (Supplemental Figure 2) for 1 hour at RT. The cells were washed 3 times with PBS, incubated with DAPI for 4 minutes, and washed with PBS. Images were taken with microscopes Zeiss Observer A1 and Z1. Image processing was performed with AxioVision software.

Total RNA isolation, reverse transcription and qRT-PCR

For DE, intestinal and lung gene analysis, cell samples were collected in 500 µL Trizol® reagent to maintain RNA integrity and stored at -80 °C before further processing. For RNA isolation, samples were thawed and 100 µL chloroform was added. The samples were centrifuged at 12,000× *g* at 4 °C, leading to phase separation. The upper aqueous phase was transferred into a new tube and total RNA was isolated by use of the NucleoSpin® RNA II Kit (Macherey-Nagel #NZ740955250). The RevertAid™ H Minus First Strand cDNA Synthesis Kit (Thermo Fisher #K1632) was used to generate cDNA from 500 ng of RNA. qPCR was performed using Absolute™ qPCR SYBR® Green Mix (Thermo Fisher #AB1159A). The cDNA was diluted 1:5 and 5uL of cDNA was added to each 25 µL reaction. Each sample was run in duplicate and Ct values were averaged for analysis. For qRT-PCR analysis FOXA2, and SOX17, CDX2, and NKX2.1, and p63 were measured in duplicates, normalized to the housekeeping genes EEF1A1 and RPL0 and run on the Mastercycler® Realplex 2 (Eppendorf). Relative gene expression was normalized to the reference genes and to undifferentiated pluripotent stem cells. Primer sequences are listed in Supplemental Table 3.

For hepatic gene analysis, RNA was isolated using the peqGOLD Total RNA Kit (Peqlab #12-6634-02) according to the manufacturer's protocol. For cDNA synthesis, the SuperScript™ First-Strand Synthesis System (Invitrogen #11904-018) was used according to the manufacturer's protocol and reverse transcribed. For qRT-PCR analysis of hepatic cells, TaqMan gene expression assays and TaqMan 2× Master Mix (Applied Biosystems #4304437) for AFP, ALB, HNF4, TTR, CK7, CK19, CYP3A4 were used. The reactions were run in triplicate on a Step One Plus Real-Time PCR System (Applied Biosystems). Glyceraldehyde-3-phosphate dehydrogenase (GAPDH) served as the endogenous control gene. Taqman sequences are listed in Supplemental Table 3.

For pancreatic gene analysis, RNA was isolated using the NucleoSpin® RNA Plus Kit (Macherey-Nagel) according to the manufacturer´s protocol. Then RNA was reverse transcribed using the RevertAid™ H Minus First Strand cDNA Synthesis Kit (Thermo Fisher #K1632) and 1 ng/µL of cDNA was applied in each 10 µL qPCR reaction performed using GoTaq® qPCR Master Mix (Promega #A6001). For pancreatic cells PDX1, SOX9, HLXB9 and HNF1B were measured in triplicates on a ViiA7 Real-Time PCR system (Applied Biosystems). Normalization was performed against the geometric mean of the housekeeping genes G6PD, TBP and TUB1A1. Primer sequences and are listed in Supplemental Table 3.

Materials

Definitive Endoderm Differentiation (CA)

Geltrex™ (Gibco #A1413202)

E8

StemPro® Accutase® (Gibco #A1110501)

10 µM Y-27632 (Tocris #1254)

CHIR99021

Activin A (PeproTech #120-14E)

Knockout™ Serum Replacement (KSR; Gibco #10828028)

125 ml Erlenmeyer flasks (Corning #431143)

Orbital shaker (Celltron, Infors HT)

50 ml conical tubes

Definitive Endoderm Differentiation (SD)

Geltrex™ (Gibco #A1413202)

E8

StemPro® Accutase® (Gibco #A1110501)

10 µM Y-27632 (Tocris #1254)

STEMDiff Definitive Endoderm Kit (STEMCELL Tech. #05115)

125 ml Erlenmeyer flasks (Corning #431143)

Orbital shaker (Celltron, Infors HT)

50 ml conical tubes

Hepatic Differentiation

Hepatocyte Culture Medium (Lonza #CC-3198)

Fibroblast Growth Factor 4 (FGF-4, Peprotech #100-31)

SB431542 (Sigma–Aldrich#S4317)

Secreted Frizzled-related Protein 5 (sFRP-5, R&D Systems #6266-SF)

Hepatocyte Growth Factor (HGF, Peprotech #100-39)

Oncostatin M (OSM; Peprotech #300-10)

Dexamethasone (Sigma Aldrich #D4902)

Y-27632 (Tocris #1254)

TrypLE (Thermo Fisher #12604013)

Matrigel (Corning #356231)

Cell culture plates

Pancreatic Differentiation

Accutase (Capricorn #ACC-1B)

Advanced RPMI 1640 medium (Gibco #12-633-012)

All-trans retinoic acid (Sigma Aldrich #302-79-4)

LDN 193189 (Selleckchem #DM-3189)

IWR-1 (Selleckchem #S7086)

FGF7 (Reliatech #100-163-L)

B27 (Gibco #17-504-044)

L-glutamine (Sigma Aldrich #G7513)

Penicillin/Streptomycin (Santa Cruz #sc-391048, Sigma Aldrich # S9137)

Y-27632 (Selleckchem #S1049)

Matrigel (Corning #354277)

Cell culture plates

Intestinal Differentiation

Geltrex™ (Gibco #A1413202)

Accutase (Gibco #A1110501)

DMEM/F12 (Gibco #11330032)

Fetal Bovine Serum (PAA #A11-101)

FGF4 (PeproTech #100-31)

CHIR99021

Penicillin/Streptomycin (Thermo Fisher 15140122)

Cell culture plates

Lung Progenitor Differentiation

Geltrex™ (Gibco #A1413202)

Accutase (Gibco #A1110501)

Knockout™ DMEM (Gibco #12660012)

Knockout™ Serum Replacement (KSR; Gibco #10828028)

L-glutamine (Gibco #25030081)

Non-essential Amino Acid Solution (Gibco #11140035)

Penicillin/Streptomycin (Gibco #25030081)

TrypLE (Thermo Fisher #12604013)

1-thioglycerol (Sigma-Aldrich #M6145)

Dorsomorphin (Sigma-Aldrich #P5499)

SB435142

Y-27632 (Tocris #1254)

IWP-2 (Tocris #3533)

BMP-4 (R&D Systems #314-BP)

CHIR99021

FGF10 (R&D Systems #345-FG)

Cell culture plates

d3 DE Analysis

CXCR4-APC (eBioscience #17-999-42)

c-Kit-PE (eBioscience #12-1178-42)

EpCam-PE (BD #347198)

Mouse IgG2a kappa Isotype APC (Thermo Fisher #17-4724-42)

Mouse IgG1 kappa Isotype Control PE (Thermo Fisher #12-4714-42)

Anti-HNF3β/FOXA2 Antibody (Merck Millipore #07-633)

human SOX17 antibody (R&D Systems #AF1924)

FACS Buffer (0.5% BSA, 3 mM EDTA, PBS)

MEDIA

DE Differentiation (CA)

d-1 CA Media

E8

10 μM Y-27632

d0 CA Media

RPMI 1640

3 μM CHIR99021

100 ng/ml Activin A

d1 CA Media

RPMI 1640

.8% KSR

100 ng/ml Activin A

d2 CA Media

RPMI 1640

8% KSR

100 ng/ml Activin A

DE Differentiation (SD)

d-1 SD Media

E8 or pluripotent stem cell media

E8 Supplement (STEMCELL Tech. #05116) (1 in 20 dilution)

10 μM Y-27632

d0 SD Media

STEMDiff™ Endoderm Basal Media

Supplement MR (1 in 100 dilution)

Supplement CJ (1 in 100 dilution)

d1-2 SD Media

STEMDiff™ Endoderm Basal Media

Supplement MR (1 in 100 dilution)

Hepatic Differentiation Media

d3 Hepatic Media

Hepatocyte Culture Medium (Lonza)

30 ng/ml FGF4

20 ng/ml BMP2

10 μM SB431542

0.5 μg/ml sFRP-5

d5 Hepatic Media

Hepatocyte Culture Medium (Lonza)

20 ng/ml HGF

d9 Hepatic Media

Hepatocyte Culture Medium (Lonza)

20 ng/ml HGF

10 ng/ml OSM

10 ng/ml dexamethasone

Pancreatic Differentiation Media

Advanced RPMI 1640

1 μM all-trans retinoic acid

0.5 μM LDN 193189

2 μM IWR-1

5 ng/ml FGF7

0.5x B27

2mM L-glutamine

1% Penicillin/Streptomycin

Lung Differentiation Media

Lung Basal Medium

Knockout™ DMEM

Knockout™ Serum Replacement (1 in
2 mM L-glutamine

1% Non-essential Amino Acid Solution

1% Penicillin/Streptomycin

0.46 mM 1-thioglycerol

Anterior Foregut Medium 1 (AFE1)

Lung Basal Medium

3 µM Dorsomorphin

10 µM SB435142

Anterior Foregut Medium 2 (AFE2)

Lung Basal Medium

2 µM IWP-2

10 µM SB435142

B4CF10 Medium

Lung Basal Medium

10 ng/ml BMP-4

3 µM CHIR99021

10 ng/ml FGF10

DE differentiation

1. At d-1, dissociate a 90% confluent flask of hPSCs with Accutase for 5 minutes.***(See NOTE)**
2. Inoculate in 125 ml Erlenmeyer flasks containing 20 ml d-1 SD or CA media at a cell density of $3.33x10^5$ cells/ml. If after 24 hours you do not see any aggregates, test higher inoculation densities to find the ideal density for aggregate formation.
3. Place flask in an incubator on an orbital shaker (Celltron, Infors HT) rotating at 70 rotations per minute (rpm).
4. After 24 hours, collect aggregates in a 50 ml conical tube and centrifuge at 300 g for 2 minutes, and add 20 ml d0 SD or CA media to the Erlenmeyer flask. After centrifugation, aspirate supernatant, collect aggregates with a serological pipette, and place in flask. Wash serological pipette and conical tube with medium to collect any remaining aggregates.
5. On d1 and d2, feed aggregates with d1 and d2 SD or CA media following step 4.
6. On day 3 of differentiation, dissociate aggregates with Accutase for 3 minutes or until you start seeing the solution become cloudy in a shaking water bath at 37°C
7. Add an equivalent amount of basis media such as DMEM/F12 supplemented with 10 µM Y-27632. Resuspend aggregates to break them apart, and then spin down at 300 g for 3 minutes.

8. Analyze cells for DE markers following flow staining protocol, and/or plate for further differentiation, and/or freeze cells with either CryoStor CS10 or conditioned DE media supplemented with 10% DMSO at 3-6x10^6 cells/vial.

***NOTE**: For the SD condition, pre-culture the pluripotent stem cells for 2 days (d-3 and d-2) prior to differentiation in E8 containing E8 Supplement provided by the SD kit.

Hepatic-like Differentiation

1. On d3, change media to d3 Hepatic Medium following step 4 of DE Differentiation.
2. On d4, dissociate cells with TrypLE for 5 minutes, and plate down cells on matrigel-coated plates at a density of 4.5x10^4/cm^2 in d3 Hepatic Medium supplemented with 10 μM Y-27632. Change media every day.
3. On d5, change media to d5 Hepatic Medium and change medium on daily basis.
4. On d9, change media to d9 Hepatic Medium on a daily basis until d14 when cells are analyzed.

Pancreatic Differentiation

1. On d3, dissociate aggregates with Accutase, and seed matrigel-coated plates at a density of 2.6x10^5 cells/cm^2 in Pancreatic Differentiation Medium supplemented with 10 μM Y-27632.
2. Feed cells every day with Pancreatic Differentiation Medium until d10 when cells are analyzed.

Intestinal Differentiation

1. On d3, dissociate aggregates with Accutase for 3 minutes in a shaking water bath at 37°C, and seed on Geltrex-coated plates at a density of 2x10^5cells/cm^2 in Intestinal Differentiation Medium supplemented with 10 μM Y-27632. Perform regular medium changes every other day until d7 when cells are analyzed.

Lung Progenitor Differentiation

1. On d3, dissociate aggregates with Accutase for 3 minutes or until solution is cloudy in a shaking water bath at 37°C.
2. Add an equivalent amount of Knockout DMEM with 10 μM Y-27632 and resuspend aggregates to break them apart.
3. Spin down aggregates at 300 g for 3 minutes, and seed cells on Geltrex-coated plates at 1x10^5 cells/cm^2 for the SD condition and 2.0x10^5 cells/cm^2 for the CA condition, in AFE1 medium supplemented with 10 μM Y-27632. You may want to plate multiple wells to test

different time points for NKX2.1 expression as peak NKX2.1 expression may vary for each cell line.

4. On d4, feed cells with fresh AFE1 medium.
5. On d6, feed cells with AFE2 medium. There is no need to feed cells on d7.
6. On d8, for the SD condition, feed cells with B4CF10 medium and feed cells every other day until day(s) of analysis. For the CA condition, dissociate cells for 5 minutes with TrypLE.
7. Stop the reaction with an equivalent amount of Knockout DMEM with 10 μM Y-27632 and spin down at 300 g for 3 minutes.
8. Plate cells on Geltrex-coated plates in B4CF10 supplemented with 10 μM Y-27632 at a 1:2 ratio. Feed cells every other day until analysis.

Thawing of dissociated DE aggregates

Only the cell density changes for this step.

1. After thawing cells and adding appropriate media (depending on which differentiation you are performing) to dilute the freezing media, spin down cells at 300 g for 3 minutes.
2. Resuspend cells in appropriate media supplemented with 10 μM Y-27632 and plate cells at the following densities:
    a. Hepatic: $4x10^4$ cells/cm^2
    b. Pancreatic: $5.26x10^5$ cells/cm^2
    c. Intestinal: $2x10^5$ cells/cm^2
    d. Lung: $1.8x10^5$ cells/cm^2 for SD
    e. Lung: $2.85x10^5$ cell/cm^2 for CA
3. Feed cells the next day with fresh medium and follow the appropriate protocol described for each lineage.

DE Differentiation in bioreactor

The DASbox Mini Bioreactor System (Eppendorf) was used. Parallel operation comprised of two independently controlled DASbox Mini bioreactor vessels for cell culture applications equipped with an eight-blade impeller (60° pitch) optimized for hPSC expansion [10]. An overhead drive allowed for smooth agitation. Sensors for pH and DO sensors were calibrated as previously described [9]. Cells were cultivated at 37°C, stirred at 60 rpm, headspace aerated with 3 sl/hour with 21% O_2 and 5% CO_2.

1. At d-1, place 100 mL of d-1 SD or CA medium in bioreactor and allow to reach 37°C.
2. Dissociate a 90% confluent flask (usually 2 T175 flasks) of hPSCs with Accutase for 5 minutes.***(See NOTE)**
3. Inoculate the 150 ml bioreactor at a density of $5x10^5$ cells/ml making sure to add enough volume to reach 150 ml of media. If after 24 hours you do not see any aggregates, test higher inoculation densities to find the ideal density for aggregate formation.

4. After 24 hours, collect aggregates in a 50 ml conical tube and centrifuge at 300 g for 2 minutes, and add 150 ml d0 SD or CA media to the bioreactor. After centrifugation, aspirate supernatant, collect aggregates with a serological pipette, and place in bioreactor. Wash serological pipette and conical tube with medium to collect any remaining aggregates.
5. On d1 and d2, feed aggregates with d1 and d2 SD or CA media following step 4.
6. On day 3 of differentiation, dissociate aggregates with Accutase for 3 minutes or until you start seeing the solution become cloudy in a shaking water bath at 37°C
7. Add an equivalent amount of basis media such as DMEM/F12 supplemented with 10 μM Y-27632. Resuspend aggregates to break them apart, and then spin down at 300 g for 3 minutes.
8. Analyze cells for DE markers following flow staining protocol, and/or plate for further differentiation, and/or freeze cells with either CryoStor CS10 or conditioned DE media supplemented with 10% DMSO at 3-6x10^6 cells/vial.

***NOTE**: For the SD condition, pre-culture the pluripotent stem cells for 2 days (d-3 and d-2) prior to differentiation in E8 containing E8 Supplement provid ed by the SD kit.
